# TET3 inhibits TGF-β1-induced epithelial-mesenchymal transition by demethylating miR-30d precursor gene in ovarian cancer cells

**DOI:** 10.1186/s13046-016-0350-y

**Published:** 2016-05-04

**Authors:** Zhongxue Ye, Jie Li, Xi Han, Huilian Hou, He Chen, Xia Zheng, Jiaojiao Lu, Lijie Wang, Wei Chen, Xu Li, Le Zhao

**Affiliations:** Center for Translational Medicine, the First Affiliated Hospital of Xi’an Jiaotong University, 277 West Yanta Road, Xi’an, Shaanxi 710061 China; Key Laboratory for Tumor Precision Medicine of Shaanxi Province, the First Affiliated Hospital of Xi’an Jiaotong University, 277 West Yanta Road, Xi’an, Shaanxi 710061 China; Department of Pathology, the First Affiliated Hospital of Xi’an Jiaotong University, 277 West Yanta Road, Xi’an, Shaanxi 710061 China; Center of Laboratory Medicine, the First Affiliated Hospital of Xi’an Jiaotong University, 277 West Yanta Road, Xi’an, Shaanxi 710061 China

**Keywords:** Ovarian cancer, Methylation, Epithelial-mesenchymal transition, TET3, TGF-β1, miR-30d

## Abstract

**Background:**

Abnormal DNA methylation/demethylation is recognized as a hallmark of cancer. TET (ten-eleven translocation) family members are novel DNA demethylation related proteins that dysregulate in multiple malignances. However, their effects on ovarian cancer remain to be elucidated.

**Methods:**

The changes of TET family members during TGF-β1-induced epithelial-mesenchymal transition (EMT) in SKOV3 and 3AO ovarian cancer cells were detected. TET3 was ectopically expressed in TGF-β1-treated ovarian cancer cells to examine its effect on TGF-β1-induced EMT phenotype. The downstream target of TET3 was further identified. Finally, the relationships of TET3 expression to clinic-pathological parameters of ovarian cancer were investigated with a tissue microarray using immunohistochemistry.

**Results:**

TET3 was downregulated during TGF-β1-initiatd epithelial-mesenchymal transition (EMT) in SKOV3 and 3AO ovarian cancer cells. Overexpression of TET3 reversed TGF-β1-induced EMT phenotypes including the expression pattern of molecular markers (E-cadherin, Vimentin, N-cadherin, Snail) and migratory and invasive capabilities of ovarian cancer cells. miR-30d was identified as a downstream target of TET3, and TET3 overexpression resumed the demethylation status in the promoter region of miR-30d precursor gene, resulting in restoration of miR-30d (an EMT suppressor of ovarian cancer cells proven in our previous study) level in TGF-β1-induced EMT. We further found that TET3 expression was decreased in ovarian cancer tissues, especially in serous ovarian cancers. The overall positivity of TET3 was inversely correlated with the grade of differentiation status of ovarian cancer.

**Conclusion:**

Our results revealed that TET3 acted as a suppressor of ovarian cancer by demethylating miR-30d precursor gene promoter to block TGF-β1-induced EMT.

**Electronic supplementary material:**

The online version of this article (doi:10.1186/s13046-016-0350-y) contains supplementary material, which is available to authorized users.

## Background

Ovarian cancer is the most lethal gynecological tumor and ranks the fifth in the cause of death for women suffered from tumor. It is estimated that there are 21,290 new ovarian cancer cases and 14,180 deaths in the United States in 2015 [1]. The poor prognosis of ovarian cancer patients is mainly attributed to cancer metastasis and recurrence. Epithelial-mesenchymal transition (EMT) is a dynamic process mediating ovarian cancer metastasis, among others. Exploration of signaling pathways involved in EMT process will shed light on the molecular mechanisms of metastasis.

EMT refers to the transformation of epithelial cells into fibroblast-like cells in physiological and pathological processes, characterized by loss of epithelial markers, acquisition of mesenchymal molecules and enhancement of cell mobility [2]. Various cytokines and growth factors, including transforming growth factor β (TGF-β), are key agents for EMT initiation and maintenance. Three isoforms of TGF-β are identified, and TGF-β1 is the most classical and frequently used EMT-inducer [3, 4].

Increasing evidence shows that aberrations in DNA methylation status are associated with tumor progression and prognosis of patients [5]. DNA methyltransferases (DNMTs) are major molecules controlling DNA methylation [6, 7]. Laterly, ten-eleven translocation (TET) family members (TET1-3) which can modify 5-methylcytosine (5-mC) by oxidation to 5-hydroxymethylcytosine (5-hmC) and further 5-formylcytosine (5-fC) and 5-carboxycytosine (5-caC) are identified and expand the understanding about mechanisms of DNA demethylation [8–10]. TETs are dysregulated in multiple malignances including breast cancer [11], hepatocellular carcinoma [11], melanoma [12] and glioma [13]. For example, decreased TET1 mRNA level is correlated with poor survival of breast cancer patients [14], and the same goes for TET2 in colorectal cancer [15].

Aberrant DNA methylation/demethylation is implicated in TGF-β1-induced EMT [16–18]. TGF-β1 triggers *TIP30* (gene coding HIV-1 Tat interactive protein 2) hypermethylation by upregulating DNMT1 and DNMT3A to induce EMT and metastasis in esophageal carcinoma [19]. However, few researches are performed to elaborate the role of TETs in TGF-β1-induced EMT. Here we report the epigenetic regulation of TET3 on miR-30d in TGF-β1-induced EMT in ovarian cancer cells, highlighting the potentiality of TET3 to be used as a prognostic biomarker or a therapeutic target for ovarian cancer.

## Methods

### Cell culture and TGF-β1 treatment

Human ovarian cancer cell line SKOV3 was obtained from the Shanghai Cell Bank of Chinese Academy of Sciences (Shanghai, China), and 3AO was from the Shandong Academy of Medical Sciences (Jinan, China). Cells were incubated in RPMI 1640 (GIBCO, Grand Island, NY USA) supplemented with 10 % newborn bovine serum (GIBCO, Grand Island, NY, USA) at 37 °C in 5 % CO_2_. When treated with 10 ng/ml TGF-β1 (PeproTech, Rocky Hill, USA), cells were maintained in media containing 1 % newborn bovine serum for indicated time before harvested.

### Quantitative real-time PCR (qRT-PCR)

Total RNA was extracted from cells using TRIzol reagent (Invitrogen, Carlsbad, CA, USA) according to the manufacturer’s instructions. Concentration and quality of total RNA were assessed by absorbance at 260 nm and the ratio of 260/280, respectively, on a UV spectrophotometer (BioRad Inc., Hercules, CA, USA). For mRNA detection, first-strand cDNA was synthesized using a PrimeScript™ RT reagent Kit with gDNA Eraser (Takara, Dalian, China). Quantitative real-time PCR was performed using a SYBR Premix Ex Taq™ II kit (Takara, Dalian, China) on a CFX96 real-time PCR system (Bio- Rad, Hercules, CA, USA). TET1, TET2 and TET3 were normalized to β-actin, while miR-30s were normalized to small nuclear U6. Relative gene expression was calculated automatically using 2^-ΔΔCt^. PCR primers for TET1, TET2, TET3 and β-actin were synthesized by Beijing Genomics Institute (Beijing, China), and primer sequences were listed in Table 1. Primers for miR-30a, 30b, 30c, 30d, 30e, and U6 reverse transcription and amplification were designed and synthesized by Ribo-Bio Co., Ltd. (Guangzhou, China).

**Table 1:** Primer sequences for real-time PCR

Genes	primer sequences (5′-3′)	Length of PCR product (bp)
TET1	F: CCCGAATCAAGCGGAAGAATA	101
	R: TACTTCAGGTTGCACGGT	
TET2	F: CTTTCCTCCCTGGAGAACAGCTC	146
	R: TGCTGGGACTGCTGCATGACT	
TET3	F: GTTCCTGGAGCATGTACTTC	93
	R: CTTCCTCTTTGGGATTGTCC	
β-actin	F: TCCCTGGAGAAGAGCTACGA	194
	R: AGCACTGTGTTGGCGTACAG	

### Western blot

Total protein was collected from cells by RIPA lysis buffer containing protease inhibitors (Roche, Indianapolis, IN, USA) and 1 mM PMSF on ice. Protein concentration was measured using the BCA-200 Protein Assay kit (Pierce, Rockford, IL, USA). After heat denaturation at 100 °C for 5 min, proteins were separated by electrophoresis on 10 % SDS–PAGE gels and then transferred onto nitrocellulose membranes (Pall Life Science, Port Washington, NY, USA). The membranes were blocked with 5 % non-fat milk at room temperature for 1 h, and then incubated overnight at 4 °C with rabbit anti-human TET3 (Abcam, 1:1000), E-cadherin (Cell Signaling Technology (CST, 1:1000), Vimentin (CST, 1:500), N-cadherin (CST, 1:1000), Snail (CST, 1:300) and mouse anti-human β-actin (CST, 1:1000). After washing with TBST, the blots were incubated with horse radish peroxidase (HRP)-conjugated goat anti-rabbit or anti-mouse IgG. Blots were visualized using ECL reagents (Pierce, Rockford, IL, USA) by a chemiluminescence imaging system (Bio-Rad, Richmond, CA, USA). The results were quantified by Image J software.

### Plasmid transient transfection

The human TET3 expression vector FH-TET3-pEF was obtained from Addgene. SKOV3 and 3AO cells were seeded into 6-well plates until 70 %-80 % confluence and transiently transfected with FH-TET3-pEF or empty vector using the X-treme GENE HP DNA Transfection Reagent (Roche, Indianapolis, IN, USA).

### miR transient transfection

miR-30d mimic and negative control were purchased from Ribo-Bio Co. Ltd. (Guangzhou, China). SKOV3 and 3AO cells were seeded into 6-well plates to reach 40 %–50 % confluence after 24 h and then transiently transfected with 100 nM miR-30d mimic or negative control using the X-treme GENE siRNA Transfection Reagent (Roche, Indianapolis, IN, USA). After 24 h of transfection, the cells were treated with 10 ng/ml TGF-β1 for another 48 h.

### Cell migration and invasion assay

After transient transfection of FH-TET3-pEF or empty vector and treatment of TGF-β1 for 48 h, cells were trypsinized and counted. A total of 1 × 10^5^ cells (for migration assay) or 4 × 10^5^ cells (for invasion assay) in 100 μl serum-free medium was added into millicells (Millipore Co., Bedford, MA, USA) without (for migration assay) or with (for invasion assay) Matrigel (Becton Dickinson Labware, Bedford, MA, USA) coated. 500 μl of medium containing 20 % newborn bovine serum was added into the bottom chambers as the chemotactic factor. After incubation for 24 h (for migration assay) or 48 h (for invasion assay) at 37 °C in 5 % CO_2_, cells remaining on the upper surface of the filter were removed using cotton swabs. Then the migrated cells were fixed using methyl alcohol and stained using 0.1 % crystal violet. Migratory (or invasive) cells were counted and averaged from images of five random fields (original magnification × 200) captured using an inverted light microscope. The mean values of three duplicate assays were used for statistical analysis.

### DNA bisulfite modification and methylation-specific PCR (MSP)

Cells treated by 10 ng/ml TGF-β1 for 48 h in 24-well plates were resuspended with cold PBS for ~6 × 10^6^/ml. DNA bisulfite modification and purification were performed using an EZ DNA methylation-Direct kit (Zymo Research Corporation, Irvine, California, USA) according to the instruction. Concentration of DNA was evaluated by absorbance at 260 nm on a UV spectrophotometer (BioRad Inc., Hercules, CA, USA). The set of primers for miR-30d gene was flanking the 3 kb region of the 5′ upstream region from the start of pre-miR-30d sequence. The primers for methylation-specific PCR were designed by MethPrimer and the sequences were as follows: methylated (M)-forward (F): 5′-TTGAGATAGGGTTTTATTTTGTCGT-3′; methylated (M)-reverse (R): 5′-TAATACATACGATCCCAACTATTCG-3′;unmethylated (U)- forward (F): 5′-TGAGATAGGGTTTTATTTTGTTGT-3′; unmethylated (U)- reverse (R): 5′-ATACATACAATCCCAACTATTCAAA-3′. DNA amplification was performed with Epi Taq HS (Takara Biotechnology Co. Ltd., Dalian, China) under the following condition: 94 °C for 5 min; 30 cycles of 94 °C for 30 s, 50 °C for 30 s, 72 °C for 30 s; and 72 °C for 10 min. The PCR products were separated by 2.0 % agarose gel electrophoresis and visualized by a chemiluminescence imaging system (Bio-Rad, Richmond, CA, USA).

### Immunohistochemistry

Human ovarian cancer tissue microarray was purchased from Shanghai SuperChip Biotech Co. Ltd. (Shanghai, China) and rabbit antibody to TET3 used for immunohistochemistry was purchased from Genetex (Alton PkwyIrvine, CA, USA). The tissue array was dewaxed in xylene, rehydrated in a descending alcohol series. Antigen retrieval was performed by heating the tissue section in 0.01 M citrate buffer (pH 6.0) in a steamer for 90 s. Detection of antigen was carried out through incubation with anti-TET3 antibody (1:250) for 2 h at room temperature, followed by incubation with HRP-labeled secondary antibody at room temperature for 30 min. Signal was generated by incubation with DAB. Slide was counterstained with hematoxylin, dehydrated in an ascending alcohol series, and mounted for analysis. Digital images were acquired using a section microscope scanner (Leica MP SCN400, German). Membrane, cytoplasm or nuclear staining was considered positive for TET3. For statistical analysis, extent (the percentage of positive cells) and intensity of staining were obtained by 2 pathologists. Intensity was semiquantitatively scored as weak (1 point), moderate (2 points), or strong (3 points). For an individual case, the immunohistochemical composite score was calculated based on the extent multiplied by the intensity score.

### Statistical analysis

The graphical presentations were performed using GraphPad Prism 5.0. Data were presented as the means ± SD and were analyzed using SPSS 22.0 software (Chicago, IL, USA). Statistical differences were tested by Chi-square test, two-tailed t-test, one-way ANOVA test or Fisher’s Exact test. Differences were considered significant at *P* <0.05 (*) or highly significant at *P* <0.001 (**).

## Results

### TET3 was reduced in TGF-β1-treated ovarian cancer cells

**Fig. 1:**
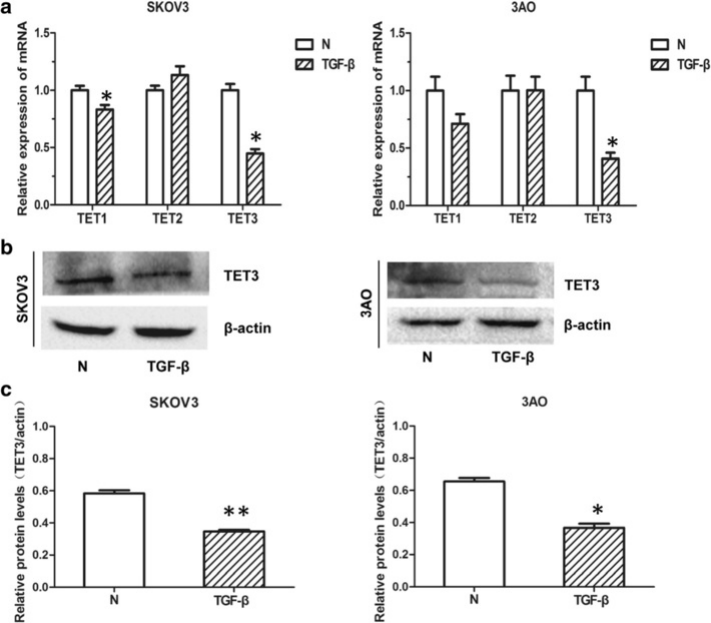
TET3 was downregulated in TGF-β1-treated ovarian cancer cells. SKOV3 and 3AO cells were maintained in 1640 medium containing 1 % newborn bovine serum with or without 10 ng/ml TGF-β1 for 48 h. **a** Quantitative real-time PCR showed that TET3 was significantly decreased at mRNA level in cells treated by TGF-β1, and TET1 was also downregulated in TGF-β1-treated SKOV3 cells. **b** Western blot results and (**c**) the quantitative analysis revealed that TET3 protein was decreased in cells stimulated by TGF-β1. All experiments were carried out in triplicate and the results were presented as means ± SD. *P < 0.05, **P < 0.01, t-test

We first examined the expression of TET family members in TGF-β1-treated ovarian cancer cells. As shown by qRT-PCR results (Fig. 1a), TET1 and TET3 were downregulated while TET2 was remained unchanged in SKOV3 and 3AO cells treated by TGF-β1. And the most significant TGF-β1-induced reduction was seen in TET3 mRNA in both cells. TET3 protein level was then detected, and consist with mRNA reduction, TET3 protein was decreased in TGF-β1-stimulated ovarian cancer cells (Fig. 1b and c), indicating the potential involvement of TET3 in TGF-β1 signaling.

### TET3 overexpression reversed TGF-β1-triggered EMT in ovarian cancer cells

**Fig. 2:**
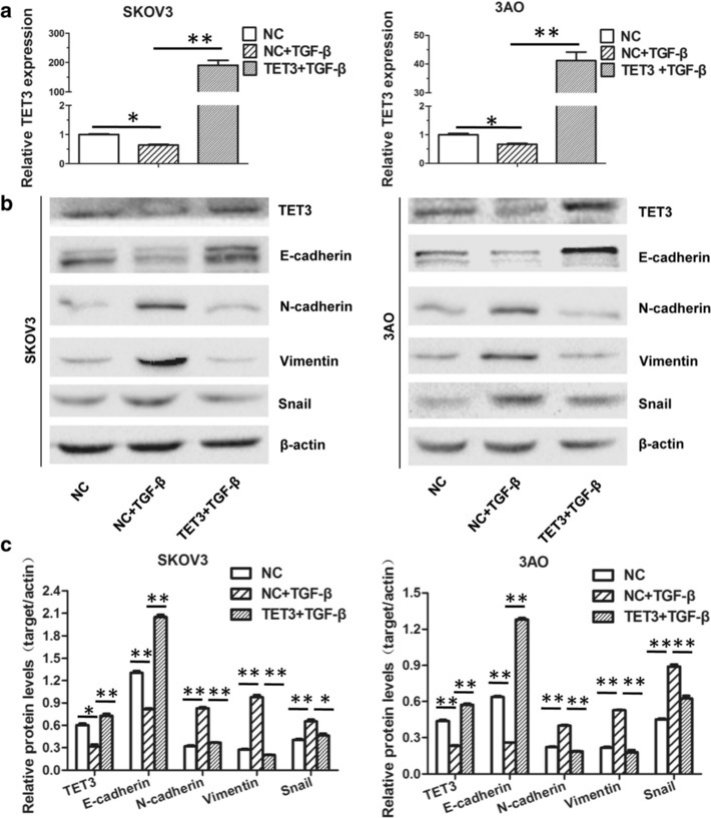
Overexpression of TET3 reversed TGF-β1-induced EMT in ovarian cancer cells. Cells were exposed to negative control, negative control plus TGF-β1 and FH-TET3-pEF transiently transfection plus TGF-β1, respectively. **a** Quantitative real-time PCR showed that transfection of FH-TET3-pEF rescued TET3 mRNA level in TGF-β1-treated cells. **b** Western blot results and (**c**) the quantitative analysis indicated that transfection of FH-TET3-pEF rescued TET3 protein level in TGF-β1-treated cells. Meanwhile, E-cadherin decrease and N-cadherin, Vimentin and Snail increase caused by TGF-β1 were reversed by TET3 overexpression. All experiments were carried out in triplicate and the values were showed as means ± SD. *P < 0.05, **P < 0.01, t-test

**Fig. 3:**
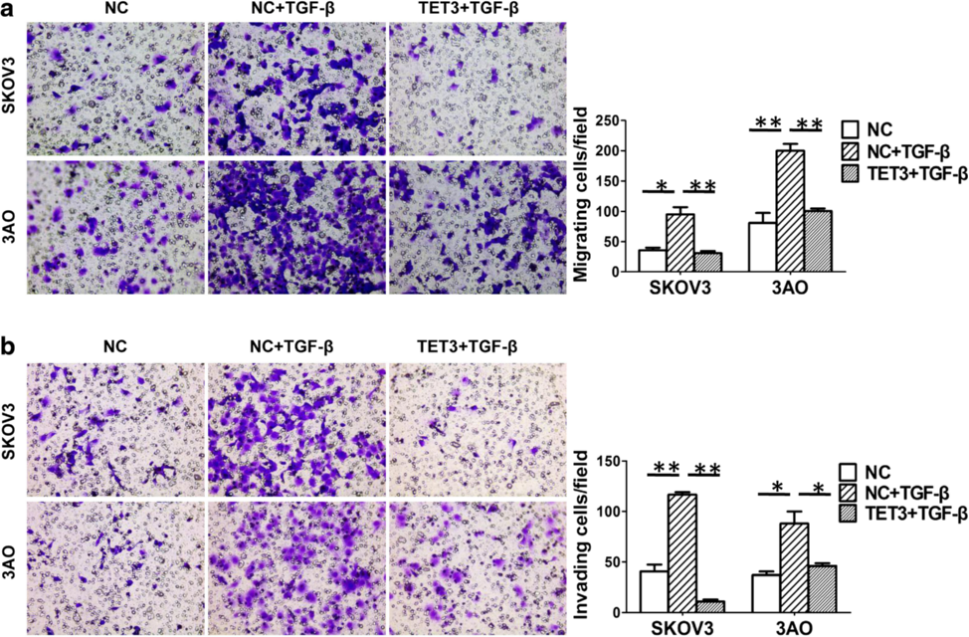
Overexpression of TET3 antagonized TGF-β1-enhanced motility and invasion of ovarian cancer cells. **a** In vitro migration assay showed that cell motility was promoted when exposed to TGF-β1, which was inhibited by TET3 overexpression (original magnification × 200). **b** In vitro invasion assay showed that TET3 overexpression quenched stimulation effect of TGF-β1 on cell invasion (original magnification × 200). All experiments were performed in triplicate and data were showed as means ± SD. *P < 0.05, **P < 0.01, t-test

Since TGF-β1 could induce EMT in ovarian cancer cells in our previous study [20], we speculated that TET3 might participate in TGF-β1-triggered EMT. To test this possibility, FH-TET3-pEF was transfected into TGF-β1-treated cells to resume the expression of TET3 (Fig. 2a). For the record, since the recombinant FH-TET3-pEF plasmid contained no fluorescence tag, the transfection efficiency was evaluated by TET3 level in TET3-transfected cells relative to negative control cells using real-time PCR and western blot (Additional file 1: Figure S1A and S1B). Restoration of TET3 antagonized TGF-β1-triggered EMT as illustrated by reversion of E-cadherin downregulation and N-cadherin, Vimentin, and Snail upregulation (Fig. 2b and c). In parallel, TET3 recovery greatly counteracted TGF-β1-stimulated enhancement of migration (Fig. 3a) and invasion (Fig. 3b) of both SKOV3 and 3AO cells. Taken together, these results showed that TET3 was a negative regulator of TGF-β1-induced EMT in ovarian cancer cells.

### TET3 upregulated miR-30d to inhibit TGF-β1-induced EMT in ovarian cancer cells

**Fig. 4:**
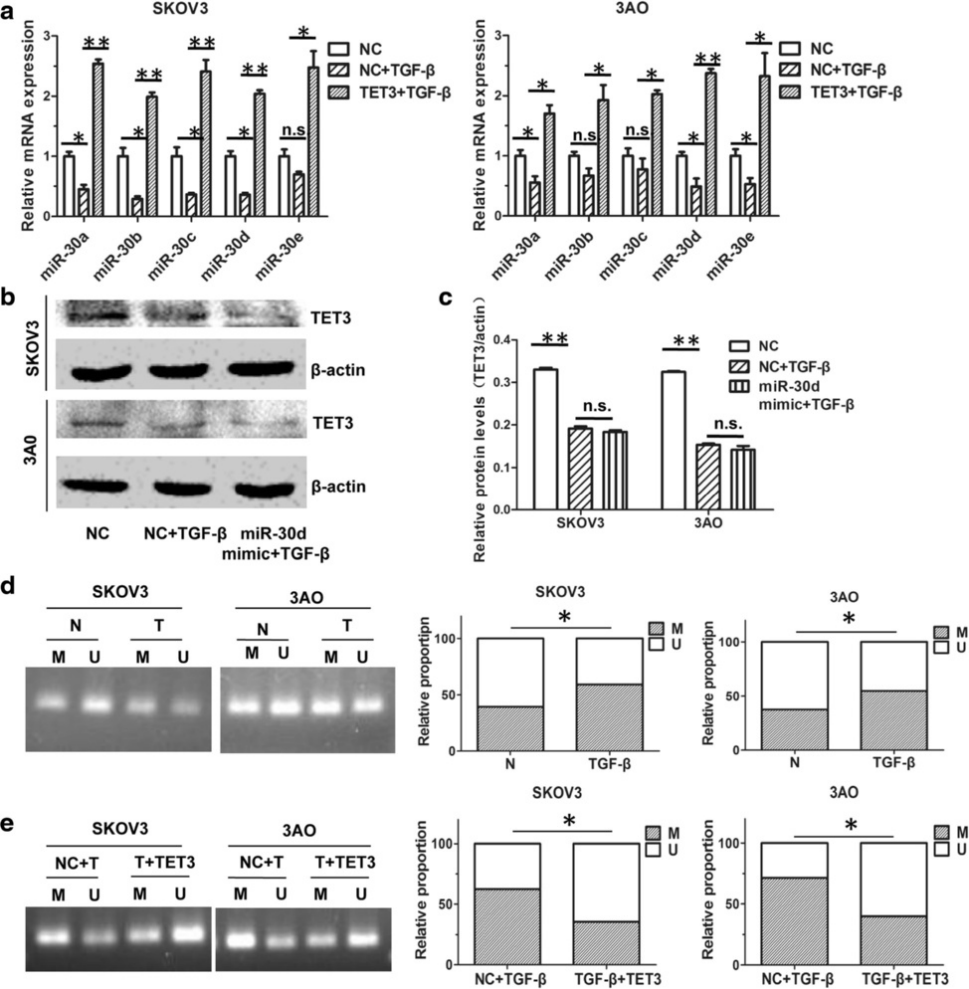
TET3 was an upstream regulator of miR-30d. **a** Quantitative real-time PCR showed that decreased miR-30s by TGF-β1 was reversed by restoration of TET3 both in SKOV3 and 3AO cells. **b** Western blot results and (**c**) the quantitative analysis indicated that resume of miR-30d could not reverse the downregulation of TET3 induced by TGF-β1 in ovarian cancer cells. **d** MSP assay proved that methylation of miR-30d precursor gene was increased in TGF-β1-treated cells. **e** Quantitative analysis of MSP results showed that methylated proportion of miR-30d precursor gene in FH-TET3-pEF transfected and TGF-β1 co-treated cells was lower than TGF-β1-treated cells. *P < 0.05, **P < 0.01, t-test

We previously found that miR-30 family members were downregulated in TGF-β1-treated ovarian cancer cells and restoration of miR-30d blocked TGF-β1-induced EMT [20]. We supposed that TET3 might oppose TGF-β1-induced EMT by increasing miR-30d. Consistent with our supposition, overexpression of TET3 resumed TGF-β1-downregulated miR-30s (Fig. 4a). In addition, co-treatment of miR-30d mimic could not reverse TGF-β1-rendered TET3 decrease (Fig. 4b and c), indicating TET3 was an upstream regulator of miR-30d, not vice versa. The MSP assay further verified that methylation of miR-30d precursor gene was increased in TGF-β1-treated cells (Fig. 4d), which was abrogated by overexpression of TET3 (Fig. 4e). Of note, TET3 overexpression alone could increase expression level of miR-30d and decrease methylation level of miR-30d precursor gene (Additional file 2: Figure S2A and S2B). These data demonstrated that TGF-β1-decreased TET3 contributed to higher methylation level of miR-30d precursor gene, subsequently caused miR-30d downregulation in ovarian cancer cells.

### TET3 was decreased in ovarian cancer tissues and negatively correlated with pathological grade

**Fig. 5:**
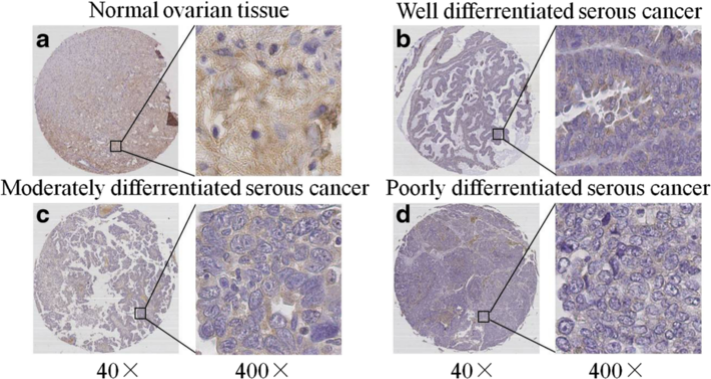
Immunostaining of TET3 in normal ovarian tissue and serous ovarian cancer. The representative photographs were taken using a section microscope scanner at 40× and 400×. **a** Moderate immunostaining of TET3 in cytoplasm of normal ovarian tissue. **b** Weak immunostaining of TET3 in cytoplasm of well differentiated serous ovarian cancer. **c** Weak positivity of TET3 in cytoplasm of moderately differentiated serous ovarian cancer. **d** Weak to negative positive staining of TET3 in cytoplasm of poorly differentiated ovarian cancer tissue

We further assessed TET3 expression with a human ovarian cancer tissue microarray, and finally obtained TET3 protein status in 67 ovarian cancer samples and 14 normal ovarian samples. TET3 mainly located in cytoplasm of both normal and tumor cells (Fig. 5). To quantify the differential TET3 expression among different subgroups, a semiquantitative scoring system was introduced by multiplying the positive extent with the intensity score. No significant difference in the overall positivity of TET3 was found between normal and cancerous tissues (*P* = 0.724). However, the immunohistochemical composite scores of cancer samples was lower than that of normal tissues (*P* = 0.0269) (Table 2). TET3 expression level in various histopathological subtypes of ovarian cancer was further analyzed. As Table 2 shown, the average score for serous ovarian cancer was significantly lower than that for normal tissues (*P* = 0.0401), while no statistic differences were found between normal tissues and other subtypes of ovarian cancer. Clinicopathological correlation analysis of TET3 level to ovarian cancer showed that the overall positivity of TET3 was inversely associated with the grade of differentiation of malignant cells (*P* = 0.024). Although no significant differences of immunohistochemical composite score were found among different differentiation status, the poorest differentiated tissues presented with lowest immunohistochemical score (*P* = 0.2409) (Table 3).

**Table 2:** Positivity and composite scores of TET3 in ovarian cancer tissues

			Immunohistochemical composite scores		
Subtypes of ovarian cancer	Overall Positivity^a^	*P* value^b^	Mean	SD	*P* value^c^
Normal (*n* = 14)	12 (85.7 %)		1.21	0.90	
Ca (*n* = 68)	52 (76.5 %)	0.724	0.72	0.72	0.0269*
EOC					
Serous cancer (*n* = 37)	29 (78.4 %)	0.707	0.71	0.70	0.0401*
Mucinous cancer (*n* = 8)	8 (100 %)	0.515	1.16	0.78	0.8932
Endometrioid cancer (*n* = 5)	4 (80 %)	1.000	1.10	1.06	0.8182
Clear cell cancer (*n* = 5)	5 (100 %)	1.000	0.86	0.67	0.4345
Germ cell tumor					
Dysgerminomas (*n* = 3)	1 (33.3 %)	0.121	0.10	0.17	0.0541
Endodermal sinus tumor (*n* = 1)	0				
Immaure teratomas (*n* = 2)	1 (50.0 %)	0.350	0.40	0.56	0.2402
Granulosa cell tumor (*n* = 3)	2 (66.7 %)	0.456	0.40	0.35	0.1508

^a^Percentage of cases with more than 5 % positive cells. ^b^Fisher’s Exact Test (two-tailed), compared with the positive percentage of normal tissues. ^c^t-test (two-tailed), compared with the composite scores of normal cases. * *P* < 0.05

**Table 3:** Clinicopathological correlation of TET3 to ovarian cancer

			Immunohistochemical composite scores		
Clinicopathological parameters of ovarian cancer	Overall Positivity^a^	*P* value	Mean	SD	*P* value
Age^b^					
< 50 (*n* = 25)	17 (68 %)	0.3793	0.65	0.77	0.9822
≥ 50 (*n* = 39)	31 (78.5 %)		0.66	0.62	
NA (*n* = 4)					
Grade^c^					
G1 (n = 7)	7 (100 %)	0.024*	0.73	0.50	0.2409
G2 (*n* = 26)	23 (88.5 %)		0.93	0.76	
G3 (*n* = 29)	18 (62.1 %)		0.59	0.76	

^a^Percentage of cases with more than 5 % positive cells. ^b^Fisher’s Exact Test (two-tailed). ^c^t-test or one-way ANOVA (two-tailed). *NA* non-available

* *P* < 0.05

## Discussion

With the deepening of studies about epigenetics and tumorigenesis, it has been admitted that abnormal DNA methylation/demethylation is a hallmark of cancer [21]. In addition to DNMTs, TETs are novel regulators of DNA methylation/demethylation status. Growing evidences suggest that deregulation of TETs and TET-mediated DNA demethylation takes part in tumor development and progression [14, 22–25].

In our study, we found that TET3 was decreased in ovarian cancer tissues, as well as in TGF-β1-treated ovarian cancer cells. Loss of TET3 might result in poorer histopathological grade in ovarian cancer patients. It was reported that TET3 was reduced in TGF-β1-activated human hepatic stellate cells (LX-2 cells), which played a critical role in liver fibrosis. Silencing of TET3 inhibited apoptosis, promoted proliferation and induced cell fibrosis in LX-2 cells by downregulating long non-coding RNA (lncRNA) HIF1A-AS1 [26]. In our experiments, TGF-β1 reduced TET3 in human ovarian cancer cells, and TET3 overexpression blocked TGF-β1-induced EMT via resuming the demethylation status of pre-miR-30d promoter region. As fibrosis was also closely connected to EMT, we speculated that TET3 could be a suppressor of EMT functioning in different tissues and EMT-associated events. In both studies, TET1 and TET2 remained almost unchanged during TGF-β1 stimulation. It might be attributed to tissue or cell specificity. Preview studies indicated that TET1 and TET2 mainly acted in embryonic stem cells (ESCs), induced pluripotent stem cells (iPSCs) and primordial germ cells (PGCs) [27–29], while TET3 was the only member identified in mouse oocytes and one-cell zygotes [30]. Although the expression pattern of TETs changed during development, differences still existed in diverse tissues and cells.

Our findings indicated that reduction of TET3 could be a result of TGF-β1 stimulation. To date, it was unclear how TGF-β1 decreased TET3. Recent studies showed that TETs were direct targets of multiple microRNAs (miRs), suggesting the proteins to be post-transcriptionally regulated by miRs [31]. miR-26, implicated in various cancers as an oncogene or tumor suppressor [32, 33], could decrease expression of all members of the TET family in vertebrates [34]. Another example was miR-29 that directly targeted TET1 in lung cancer cells [35], and all TET family members in human dermal fibroblasts and vascular smooth muscle cells [36]. Interestingly, miR-29 was a critical mediator in TGF-β/Smad signaling [37]. Thus, we presumed that TET3 reduction in our model could be a result of miR dysregulation. Nevertheless, TET3 could be also controlled by DNA methylation/demethylation, as found in clinical samples [15]. Illumination of the molecular underpinnings of TGF-β-induced TET3 reduction would contribute to understanding the regulatory network in TGF-β-stimulated EMT.

## Conclusions

Our results indicated that TET3 declined in TGF-β1 stimulation and TET3 overexpression inhibited TGF-β1-induced EMT and EMT-mediated metastasis of SKOV3 and 3AO cells by demethylating miR-30d precursor gene, indicating a novel mechanism of epigenetic regulation in ovarian cancer. Targeting the TGF-β1-TET3-miR-30d signaling axis might be a promising therapeutic strategy for ovarian cancer treatment.

## Additional files

Additional file 1: Figure S1. Transfection efficiency of recombinant expression plasmid for TET3. **a** Quantitative real-time PCR showed that TET3 was increased by about 100 times in TET3-transfected SKOV3 cells relative to negative control cells. **b** Western blot results and the quantitative analysis revealed that TET3 protein was increased by about 3 times in TET3-transfected SKOV3 cells relative to negative control cells. All experiments were carried out in triplicate and the results were presented as means ± SD. **P* < 0.05, t-test. (JPG 132 kb) Additional file 2: Figure S2. The effect of TET3 overexpression on miR-30d. **a** Quantitative real-time PCR showed that miR-30d was increased by ectopic expression of TET3 in SKOV3 cells. **b** MSP assay found that methylation of miR-30d precursor gene was decreased in TET3-overexpressed SKOV3 cells. (JPG 123 kb)

## References

[1] Siegel RL, Miller KD, Jemal A (2015). Cancer statistics, 2015. CA: A Cancer Journal for Clinicians.

[2] Kalluri R, Weinberg RA (2009). The basics of epithelial-mesenchymal transition. J Clin Invest..

[3] Katz LH, Li Y, Chen JS, Munoz NM, Majumdar A, Chen J, Mishra L (2013). Targeting TGF-beta signaling in cancer. Expert Opin Ther Targets..

[4] Kaufhold S, Bonavida B (2014). Central role of Snail1 in the regulation of EMT and resistance in cancer: a target for therapeutic intervention. J Exp Clin Cancer Res..

[5] Dawson MA, Kouzarides T (2012). Cancer epigenetics: from mechanism to therapy. Cell..

[6] Holliday R, Pugh JE (1975). DNA modification mechanisms and gene activity during development. Science..

[7] Riggs AD (1975). X inactivation, differentiation, and DNA methylation. Cytogenet Cell Genet..

[8] Tahiliani M, Koh KP, Shen Y, Pastor WA, Bandukwala H, Brudno Y, Agarwal S, Iyer LM, Liu DR, Aravind L, Rao A (2009). Conversion of 5-methylcytosine to 5-hydroxymethylcytosine in mammalian DNA by MLL partner TET1. Science..

[9] Ito S, D’Alessio AC, Taranova OV, Hong K, Sowers LC, Zhang Y (2010). Role of Tet proteins in 5mC to 5hmC conversion, ES-cell self-renewal and inner cell mass specification. Nature..

[10] Ito S, Shen L, Dai Q, Wu SC, Collins LB, Swenberg JA, He C, Zhang Y (2011). Tet proteins can convert 5-methylcytosine to 5-formylcytosine and 5-carboxylcytosine. Science..

[11] Yang H, Liu Y, Bai F, Zhang JY, Ma SH, Liu J, Xu ZD, Zhu HG, Ling ZQ, Ye D, Guan KL, Xiong Y (2013). Tumor development is associated with decrease of TET gene expression and 5-methylcytosine hydroxylation. Oncogene..

[12] Lian CG, Xu Y, Ceol C, Wu F, Larson A, Dresser K, Xu W, Tan L, Hu Y, Zhan Q, Lee CW, Hu D, Lian BQ, Kleffel S, Yang Y, Neiswender J, Khorasani AJ, Fang R, Lezcano C, Duncan LM, Scolyer RA, Thompson JF, Kakavand H, Houvras Y, Zon LI, Mihm MC, Kaiser UB, Schatton T, Woda BA, Murphy GF, Shi YG (2012). Loss of 5-hydroxymethylcytosine is an epigenetic hallmark of melanoma. Cell..

[13] Muller T, Gessi M, Waha A, Isselstein LJ, Luxen D, Freihoff D, Freihoff J, Becker A, Simon M, Hammes J, Denkhaus D, zur Muhlen A, Pietsch T (2012). Nuclear exclusion of TET1 is associated with loss of 5-hydroxymethylcytosine in IDH1 wild-type gliomas. Am J Pathol..

[14] Hsu CH, Peng KL, Kang ML, Chen YR, Yang YC, Tsai CH, Chu CS, Jeng YM, Chen YT, Lin FM, Huang HD, Lu YY, Teng YC, Lin ST, Lin RK, Tang FM, Lee SB, Hsu HM, Yu JC, Hsiao PW, Juan LJ (2012). TET1 suppresses cancer invasion by activating the tissue inhibitors of metalloproteinases. Cell Rep..

[15] Rawluszko-Wieczorek AA, Siera A, Horbacka K, Horst N, Krokowicz P, Jagodzinski PP (2015). Clinical significance of DNA methylation mRNA levels of TET family members in colorectal cancer. J Cancer Res Clin Oncol..

[16] Zhang Q, Chen L, Helfand BT, Jang TL, Sharma V, Kozlowski J, Kuzel TM, Zhu LJ, Yang XJ, Javonovic B, Guo Y, Lonning S, Harper J, Teicher BA, Brendler C, Yu N, Catalona WJ, Lee C (2011). TGF-beta regulates DNA methyltransferase expression in prostate cancer, correlates with aggressive capabilities, and predicts disease recurrence. PLoS One..

[17] Cardenas H, Vieth E, Lee J, Segar M, Liu Y, Nephew KP, Matei D (2014). TGF-beta induces global changes in DNA methylation during the epithelial-to-mesenchymal transition in ovarian cancer cells. Epigenetics..

[18] Kogure T, Kondo Y, Kakazu E, Ninomiya M, Kimura O, Shimosegawa T (2014). Involvement of miRNA-29a in epigenetic regulation of transforming growth factor-beta-induced epithelial-mesenchymal transition in hepatocellular carcinoma. Hepatol Res..

[19] Bu F, Liu X, Li J, Chen S, Tong X, Ma C, Mao H, Pan F, Li X, Chen B, Xu L, Li E, Kou G, Han J, Guo S, Zhao J, Guo Y (2015). TGF-beta1 induces epigenetic silence of TIP30 to promote tumor metastasis in esophageal carcinoma. Oncotarget..

[20] Ye Z, Zhao L, Li J, Chen W, Li X (2015). miR-30d Blocked Transforming Growth Factor beta1-Induced Epithelial-Mesenchymal Transition by Targeting Snail in Ovarian Cancer Cells. Int J Gynecol Cancer..

[21] Rengucci C, De Maio G, Casadei Gardini A, Zucca M, Scarpi E, Zingaretti C, Foschi G, Tumedei MM, Molinari C, Saragoni L, Puccetti M, Amadori D, Zoli W, Calistri D (2014). Promoter methylation of tumor suppressor genes in pre-neoplastic lesions; potential marker of disease recurrence. J Exp Clin Cancer Res..

[22] Ko M, Huang Y, Jankowska AM, Pape UJ, Tahiliani M, Bandukwala HS, An J, Lamperti ED, Koh KP, Ganetzky R, Liu XS, Aravind L, Agarwal S, Maciejewski JP, Rao A (2010). Impaired hydroxylation of 5-methylcytosine in myeloid cancers with mutant TET2. Nature..

[23] Abdel-Wahab O, Mullally A, Hedvat C, Garcia-Manero G, Patel J, Wadleigh M, Malinge S, Yao J, Kilpivaara O, Bhat R, Huberman K, Thomas S, Dolgalev I, Heguy A, Paietta E, Le Beau MM, Beran M, Tallman MS, Ebert BL, Kantarjian HM, Stone RM, Gilliland DG, Crispino JD, Levine RL (2009). Genetic characterization of TET1, TET2, and TET3 alterations in myeloid malignancies. Blood..

[24] Takayama K, Misawa A, Suzuki T, Takagi K, Hayashizaki Y, Fujimura T, Homma Y, Takahashi S, Urano T, Inoue S (2015). TET2 repression by androgen hormone regulates global hydroxymethylation status and prostate cancer progression. Nat Commun..

[25] Lorsbach RB, Moore J, Mathew S, Raimondi SC, Mukatira ST, Downing JR (2003). TET1, a member of a novel protein family, is fused to MLL in acute myeloid leukemia containing the t (10;11) (q22;q23). Leukemia..

[26] Zhang QQ, Xu MY, Qu Y, Hu JJ, Li ZH, Zhang QD, Lu LG (2014). TET3 mediates the activation of human hepatic stellate cells via modulating the expression of long non-coding RNA HIF1A-AS1. Int J Clin Exp Pathol..

[27] Guibert S, Forne T, Weber M (2012). Global profiling of DNA methylation erasure in mouse primordial germ cells. Genome Res..

[28] Costa Y, Ding J, Theunissen TW, Faiola F, Hore TA, Shliaha PV, Fidalgo M, Saunders A, Lawrence M, Dietmann S, Das S, Levasseur DN, Li Z, Xu M, Reik W, Silva JC, Wang J (2013). NANOG-dependent function of TET1 and TET2 in establishment of pluripotency. Nature..

[29] Vincent JJ, Huang Y, Chen PY, Feng S, Calvopina JH, Nee K, Lee SA, Le T, Yoon AJ, Faull K, Fan G, Rao A, Jacobsen SE, Pellegrini M, Clark AT (2013). Stage-specific roles for tet1 and tet2 in DNA demethylation in primordial germ cells. Cell Stem Cell..

[30] Iqbal K, Jin SG, Pfeifer GP, Szabo PE (2011). Reprogramming of the paternal genome upon fertilization involves genome-wide oxidation of 5-methylcytosine. Proc Natl Acad Sci U S A..

[31] Cheng J, Guo S, Chen S, Mastriano SJ, Liu C, D’Alessio AC, Hysolli E, Guo Y, Yao H, Megyola CM, Li D, Liu J, Pan W, Roden CA, Zhou XL, Heydari K, Chen J, Park IH, Ding Y, Zhang Y, Lu J (2013). An extensive network of TET2-targeting MicroRNAs regulates malignant hematopoiesis. Cell Rep..

[32] Zeitels LR, Acharya A, Shi G, Chivukula D, Chivukula RR, Anandam JL, Abdelnaby AA, Balch GC, Mansour JC, Yopp AC, Richardson JA, Mendell JT (2014). Tumor suppression by miR-26 overrides potential oncogenic activity in intestinal tumorigenesis. Genes Dev..

[33] Huse JT, Brennan C, Hambardzumyan D, Wee B, Pena J, Rouhanifard SH, Sohn-Lee C, le Sage C, Agami R, Tuschl T, Holland EC (2009). The PTEN-regulating microRNA miR-26a is amplified in high-grade glioma and facilitates gliomagenesis in vivo. Genes Dev..

[34] Fu X, Jin L, Wang X, Luo A, Hu J, Zheng X, Tsark WM, Riggs AD, Ku HT, Huang W (2013). MicroRNA-26a targets ten eleven translocation enzymes and is regulated during pancreatic cell differentiation. Proc Natl Acad Sci U S A..

[35] Morita S, Horii T, Kimura M, Ochiya T, Tajima S, Hatada I (2013). miR-29 represses the activities of DNA methyltransferases and DNA demethylases. Int J Mol Sci..

[36] Zhang P, Huang B, Xu X, Sessa WC (2013). Ten-eleven translocation (Tet) and thymine DNA glycosylase (TDG), components of the demethylation pathway, are direct targets of miRNA-29a. Biochem Biophys Res Commun..

[37] Zhang Y, Huang XR, Wei LH, Chung AC, Yu CM, Lan HY (2014). miR-29b as a therapeutic agent for angiotensin II-induced cardiac fibrosis by targeting TGF-beta/Smad3 signaling. Mol Ther..

