# Supplementary figures and images for: TET3 inhibits TGF-β1-induced epithelial-mesenchymal transition by demethylating miR-30d precursor gene in ovarian cancer cells
Source: J Exp Clin Cancer Res. 2016 May 4;35:72. doi: 10.1186/s13046-016-0350-y (PMC4855705; doi:10.1186/s13046-016-0350-y)

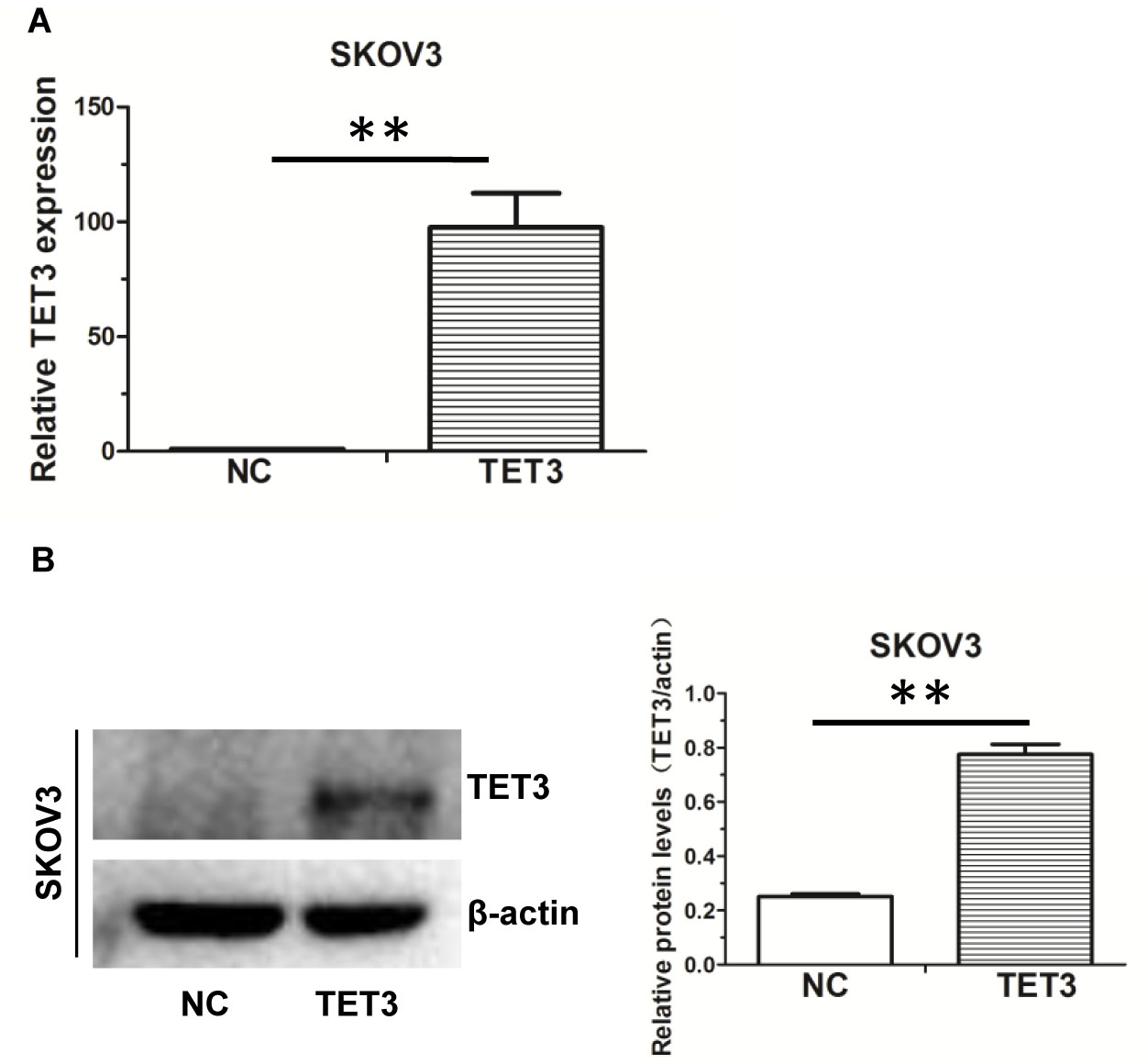

Supplement: Additional file 1: Figure S1. — Transfection efficiency of recombinant expression plasmid for TET3. a Quantitative real-time PCR showed that TET3 was increased by about 100 times in TET3-transfected SKOV3 cells relative to negative control cells. b Western blot results and the quantitative analysis revealed that TET3 protein was increased by about 3 times in TET3-transfected SKOV3 cells relative to negative control cells. All experiments were carried out in triplicate and the results were presented as means ± SD. *P < 0.05, t-test. (JPG 132 kb) [file 13046_2016_350_MOESM1_ESM.jpg]

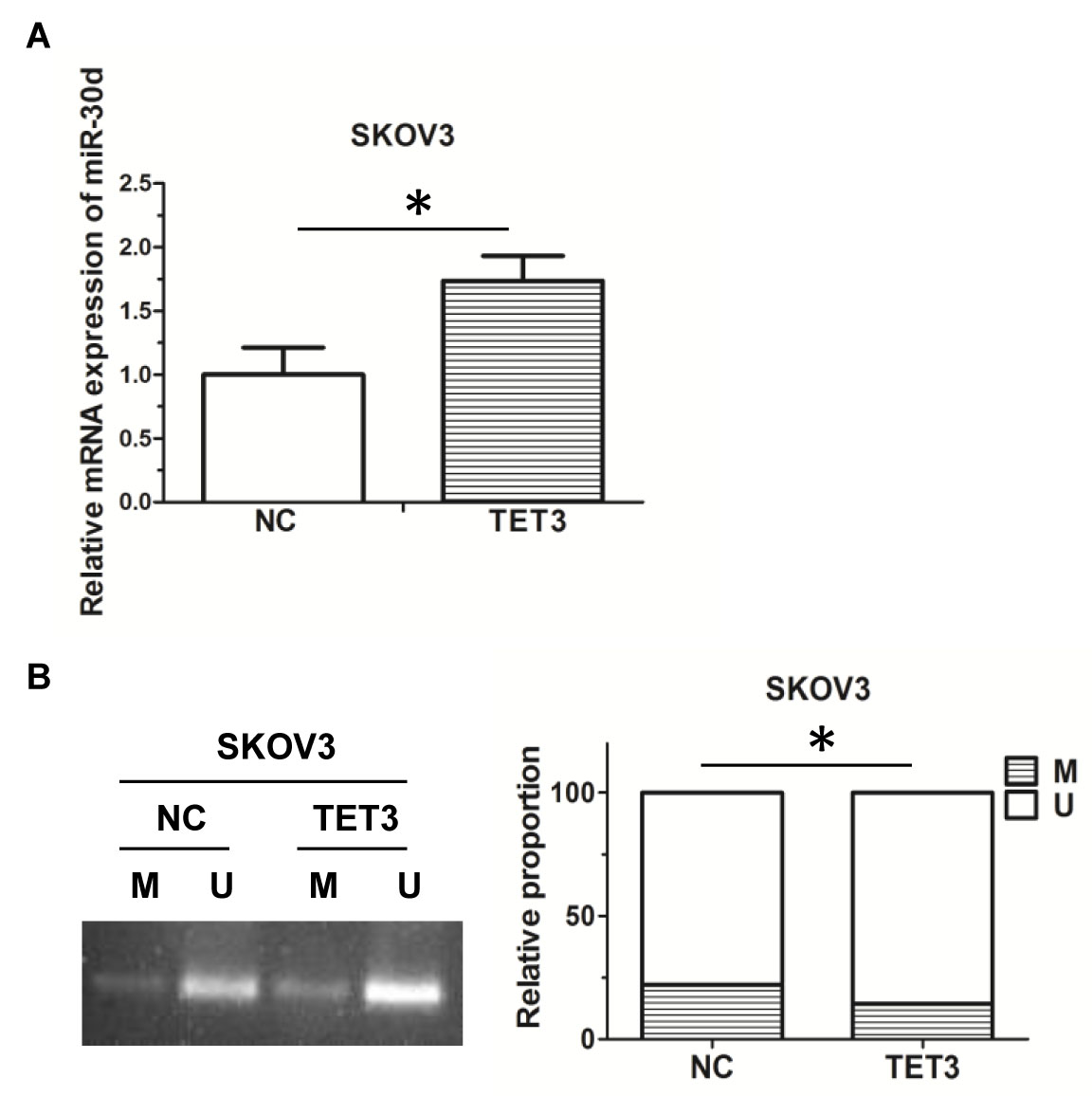

Supplement: Additional file 2: Figure S2. — The effect of TET3 overexpression on miR-30d. a Quantitative real-time PCR showed that miR-30d was increased by ectopic expression of TET3 in SKOV3 cells. b MSP assay found that methylation of miR-30d precursor gene was decreased in TET3-overexpressed SKOV3 cells. (JPG 123 kb) [file 13046_2016_350_MOESM2_ESM.jpg]
